# From the body to the mind: interoception and sense of agency as mechanisms of depression reduction in the Body–Mind Axial Awareness (BMAA)

**DOI:** 10.3389/fpsyg.2026.1755698

**Published:** 2026-04-07

**Authors:** Yunn-Wen Lien, Shan-Chuan Teng, Li-Yun Wei

**Affiliations:** Department of Psychology, National Taiwan University, Taipei, Taiwan

**Keywords:** depression, embodied practices, emotion regulation, interoception, mindfulness, sense of agency, wellbeing

## Abstract

**Introduction:**

Psychological wellbeing among young adults has declined globally, along with rising levels of depression, creating an educational and societal crisis and highlighting the urgent need for school-based mental health interventions. In response, the Body–Mind Axial Awareness (BMAA) method—an embodied body–mind enhancement program rooted in East Asian self-cultivation traditions—was developed at National Taiwan University to promote students’ mental resilience and wellbeing.

**Methods:**

This study examined the effects and mechanisms of a 10-week BMAA course on depressive tendencies (measured by the Beck Depression Inventory), interoceptive sensibility [measured by the Multidimensional Assessment of Interoceptive Awareness (MAIA)], and sense of agency (measured by the Sense of Agency Scale) in university students. To test these aims, a pre–post design with an active control group was used (BMAA: *n* = 50; control: *n* = 21).

**Results:**

The findings showed that BMAA participants experienced significant reductions in depressive tendencies, improvements across all dimensions of interoceptive sensibility, and decreases in negative sense of agency (SoNA) compared with the control group. Mediation analyses further revealed a serial pathway in which enhancements in the Not-Distracting, Attention Regulation, and Trusting dimension of interoception led to reductions in SoNA, which in turn contributed to decreased depressive tendencies. Additionally, independent mediation pathways involving the Not-Worrying dimension and SoNA alone were also significant.

**Discussion:**

To our knowledge, this is the first evidence that interoception and the sense of agency play crucial, sequential roles in how embodied practices like 2 BMAA support emotion regulation. BMAA not only offers a feasible method for promoting students’ mental health and resilience but also shows promising potential for broader clinical use in the future.

## 1 Introduction

Psychological well-being, or happiness, is widely regarded as a core indicator of optimal psychological experiences and functioning, with established links to both mental and physical health (e.g., Diener, 1984; Ryff, 1989; Csikszentmihalyi, 1990; Ryan and Deci, 2001; Ryff, 2013). Over the past decade, however, extensive research and surveys have revealed a concerning trend: a significant decline in well-being and a corresponding rise in mental distress, such as depression and anxiety, among young adults under 25, particularly in developed countries (e.g., Auerbach et al., 2016; Duffy et al., 2020; Auty et al., 2022; McGorry et al., 2023; Udupa et al., 2023; Dykxhoorn et al., 2024; Blanchflower et al., 2025a,b; Twenge and Blanchflower, 2025). Recognizing this as a pressing educational and societal crisis, researchers and higher education institutions have called for increased attention and investment to improve mental health in educational settings (e.g., CCVPS, 2015; Thorley, 2017; Universities UK, 2018).

In response to this global mental health crisis, a body–mind enhancement program—the Body–Mind Axial Awareness (BMAA) method—was developed over the past decade by the first author and her research team as a regular course at National Taiwan University. This method can be seen as a modern psychological adaptation of the East Asian embodied self-cultivation tradition, or *xiū-shēn* (修身, literally “cultivating the body”). In this study, we focus on two main aims. First, we examine the effectiveness of BMAA in reducing mental distress, specifically the tendency toward depression among university students. Second, we explore whether any reduction in depression occurs through a serial mediating pathway involving training-related improvements in two potential mediators: interoception and the sense of agency (SoA). Interoception refers to the process of sensing, interpreting, and integrating signals from inside the body (Khalsa et al., 2018; Chen et al., 2021), while SoA is the subjective feeling of being in control of one’s actions and their outcomes (Gallagher, 2000, 2012).

In the following, we will first review the role of interoception in downregulating emotions. Next, we will argue that SoA is a plausible serial mediator through which specific aspects of interoceptive sensibility help reduce depressive tendencies, especially within a dynamic embodied approach such as the BMAA method. Finally, we will introduce the cultural background and principles of BMAA, followed by the rationale for our short-term intervention study, which uses a pre- and post-test design with an active control group.

The close link between emotion and physiological arousal has been recognized since the earliest psychological theories of emotion over a century ago (James, 1884/1948; Lange, 1885/1922). Recent research further shows that our bodily senses—internal sensations from the motor and visceral systems, collectively termed interoception—are crucial for emotional processing and cognitive functions (e.g., Damasio, 1994; Critchley and Harrison, 2013; Barrett, 2017). This perspective highlights that body awareness is not merely a byproduct of emotion but an embodied process for understanding and regulating emotions (e.g., Craig, 2002; Barrett and Simmons, 2015).

For instance, Füstös et al. (2013) found that higher trait interoceptive awareness is linked to stronger, more efficient neural responses in brain regions associated with interoception during cognitive reappraisal, a top-down emotional regulation strategy. Additionally, those with high awareness perceive themselves as more successful at modulating their arousal levels. Greater self-reported interoceptive awareness, emotional granularity, and psychological well-being have also been found to be pairwise associated (e.g., Kashdan et al., 2015; Ventura-Bort et al., 2021; Gross et al., 2025). Consequently, enhancing interoceptive awareness has become a central goal and a key mechanism in interventions using embodied practices—such as mindfulness, biofeedback, and body-centered trauma therapies—to alleviate mental distress (Hölzel et al., 2011; van der Kolk, 2014; Wareing et al., 2024).

However, evidence suggests the relationship between interoceptive processing and mental distress is more complex than initially conceptualized, characterized by two primary inconsistencies. First, empirical findings remain inconsistent across different assessment modalities. Objective interoceptive accuracy, as assessed by cardiac or respiratory detection tasks, yields unstable correlations with distress (Adams et al., 2022; Desmedt et al., 2022; van der Does et al., 2000; Wu et al., 2021). Similarly, subjective sensibility reveals divergent patterns across its dimensions. On the commonly used MAIA scale, for instance, the “Trusting” and “Self-Regulation” dimensions typically align with better mental health, whereas “Not-Distracting” presents a context-dependent paradox—associating with higher depression in some studies (Dunne et al., 2021; Solano López and Moore, 2019) yet facilitating recovery in clinical pain populations (de Jong et al., 2016).

Second, while some dimensions of interoceptive sensibility facilitate the development of metacognitive skills that underpin the efficacy of mindfulness-based interventions, others do not. Research indicates that the MAIA subscales “Attention Regulation,” “Self-Regulation,” “Body Listening,” and “Trusting” each contribute to improved decentering from thoughts and emotions, thereby alleviating depressive symptoms (Fissler et al., 2016). Furthermore, expanded interoceptive awareness may enhance top-down regulation by facilitating cognitive reappraisal, which in turn increases positive emotional experiences (Garland et al., 2017).

In summary, these findings demonstrate that the impact of interoception depends heavily on the specific dimensions and measurement methods used. Current models, especially those rooted in mindfulness, emphasize how certain interoceptive dimensions support the development of metacognitive or cognitive reframing skills (e.g., Decentering and Reappraisal), indicating a predominantly top-down pathway.

Compared with traditional mindfulness training, our method—BMAA—is a more dynamic, bottom-up, and physically active embodied intervention. We hypothesize that its positive effects on mental distress, especially depression, may involve a more bodily-rooted pathway, possibly by enhancing SoA. One of our study’s goals was to examine a new mechanistic chain: whether this embodied approach improves specific aspects of interoceptive sensibility, leading to increased SoA and ultimately reducing depressive tendencies. The following paragraphs explain why SoA is a plausible candidate, given its embodied nature and its connection to interoception and psychopathology.

As mentioned, SoA is often described as the alignment between a person’s intention and action (Gallagher, 2012) or as the feeling of being in control of one’s own actions (van der Kolk, 2014). It is usually viewed as a subjective feeling deeply rooted in embodied experience. As noted, constantly monitoring feedback from the body is necessary to align one’s actions with intentions, which is a primary source of agency (Bermúdez, 2018). Additionally, a person’s subjective judgments about their bodily abilities and status significantly influence their SoA (de Vignemont, 2018). From a developmental perspective, SoA may originate from successful experiences in responding to interoceptive states through action (van der Kolk, 2014; Deans et al., 2015; Farb et al., 2015). For example, infants develop a sense of control when their cries reliably elicit responses from caregivers. The joint development of interoception and motor control further underscores the strong link between interoception and SoA (Musculus et al., 2021).

Although limited, some studies empirically examine the link between interoception and SoA. To our knowledge, two recent studies show that better interoception, as measured by self-reported interoceptive sensibility or cardiac interoceptive accuracy, tends to correlate with higher SoA, as assessed by a subjective scale or a specific objective index (Stern et al., 2020; Koreki et al., 2022). These findings suggest that reliable interoceptive perception or sensibility is essential for experiencing control over one’s actions.

Furthermore, a disrupted or diminished SoA is a common feature across various mental health conditions (e.g., Gallagher and Trigg, 2016; Tapal et al., 2017; Rabellino et al., 2018; Colle et al., 2020; Colle et al., 2023; Mehta et al., 2023). For instance, the severity of depression has been shown to be inversely related to both self-rated and objectively measured agency (Vogel et al., 2024). This relationship suggests that mental health relies not only on effective interoception but also on experiencing oneself as an agent capable of regulating bodily sensations through action.

Although empirical links have been established between interoception and SoA, and between SoA and psychopathology, the underlying mechanisms in intervention settings remain largely unexplored. In other words, few, if any, studies have examined whether improved SoA mediates the reduction in mental distress after an embodied intervention. To address this research gap, the present study explicitly tests whether improved SoA, facilitated by improved interoception, mediates a reduction in depressive tendencies following our embodied training—BMAA. Departing from models that view the body primarily as a source of interoceptive signals for top-down regulation, we conceptualize the body as a “dwelling” or “carrier” of unresolved physiological tensions and emotions. To establish the conceptual foundation for this intervention, we first trace its traditional roots and then elucidate the embodied principles and components that underpin its approach to emotion regulation.

BMAA adopts a holistic perspective derived from East Asian traditions, emphasizing a transformative process toward a more harmonious state of being. This ideal state traces back to the ancient pursuit of the “Oneness of Heaven and the Human Self,” originally cultivated through *Ya-Yue* (ritual dance and music) in sacrificial ceremonies to facilitate a transcendent connection with the cosmos (Lien et al., 2019; Yu, 2014). Such an optimal mind–body state is marked by the absence of lingering emotional or somatic tensions, manifesting as a congruence between inner tranquility and a fluid, unobstructed physical system. Inner tranquility, or *jìng* (靜), denotes a clear, peaceful state of consciousness free from the disturbance of reactive emotions, desires, or cognitive prejudices (Yang, 1996); whereas a fluid body is flexible, grounded, and aligned along its central axis—a perceived vertical line extending from the perineum to the vertex (for a more detailed description of the central axis, see Teng and Lien, 2016).

In modern anatomical terms, this central axis may involve maintaining dynamic equilibrium within the fascial matrix—a continuous web that supports and interconnects the internal organs and the musculoskeletal framework (Stecco et al., 2011). Based on our analysis and observations, the central axis emerges as a felt sense originating from the base of the pelvic cavity once a degree of “sinking relaxation” and upright alignment is achieved. For many beginners, this felt sense is often fragmented rather than continuous, reflecting some localized blockages.

Traditionally, this alignment is thought to facilitate the transmission of vital substances and energy (*Chi*). Furthermore, BMAA redefines the axis as a psychological anchor, allowing practitioners to gently “park” their attention and settle the mind. Notably, life stressors—including aging, injuries, habitual postures, and suppressed emotions—can cause chronic stiffness or somatic rigidity, leading to a deviation from our original optimal state (Chen, 2011; Lien et al., 2019).

Building on this foundation, we further posit that physical rigidity maladaptively influences emotion regulation through four interrelated pathways. First, because fascia is rich in receptors (Schleip and Jäger, 2012), chronic stiffness may induce interoceptive distortion and disrupt signal clarity, impairing the delicate awareness needed for early emotional detection and downregulation. Second, rigidity constricts the physiological pathways that allow tension to dissipate naturally, trapping emotion-driven energy and exacerbating further somatic rigidity. Third, it lowers the threshold for emotional reactions. Encapsulated negative energies can be triggered by associated cues, increasing the risk of overreaction and internal instability, characterized by a loss of *jìng*. Fourth, body rigidity limits the behavioral flexibility needed to manage emotions or stress, further increasing stress levels and reducing one’s perceived sense of agency. While current literature has begun to link bodily rigidity to depression (Michalak et al., 2022) and myofascial intervention to reduced anxiety (Gozalo-Pascual et al., 2023), our model further elucidates the specific psychosomatic processes that mediate these relationships.

While early Confucianism and Taoism reinterpreted these ancient practices as a path to self-cultivation and self-transcendence (Hsu, 1966; Yu, 2014), subsequent mainstream traditions often prioritized moral conduct, leaving the specific somatic foundations implicit or dispersed across specialized fields such as martial arts. Drawing on Chen’s (2011) phenomenological deconstruction of *Ya-Yue* dance, BMAA integrates and reinterprets these ancient roots into a structured somatic practice tailored for modern psychological needs. Specifically, BMAA offers a bottom-up pathway to enhance psychological resilience by helping practitioners release “stuck” emotions and untangle bodily tension, restoring a free-flowing body–mind state.

In the following section, we detail the somatic movement component, its mechanical principles, and the psychological component of BMAA. The BMAA method centers on a set of structured, coherent dynamic somatic exercises. The main feature of these practices involves subtle movements characterized by minimal volitional control over superficial muscles. To keep superficial muscles relaxed while moving the extremities, practitioners learn to use supporting or resistance surfaces—such as a wall, floor, or tools—as physical fulcrums, enabling movement with minimal effort and intention. These exercises are designed to engage various body parts and fascial pathways, progressing systematically from highly supported lying positions to standing postures, primarily focusing on the extremities before gradually integrating them with the central trunk. This systematic approach is intended to gradually loosen physical and psychological obstacles from the periphery to the core and from the superficial to the deep layers relative to the central axis. Through repeated practice, participants learn to be aware of subtle inner forces—likely changes in fascial tension—that arise from supportive surfaces and travel along the axis to a specific extremity. The smoothness and clarity of this inner force indicate the degree of fluidity, providing feedback on one’s progress. These same principles and mental state are reinforced through diverse movement practices throughout the course.

Furthermore, BMAA uniquely includes instructions to facilitate or cultivate the required mental state and attitude. First, practitioners are reminded to approach the practice as a non-striving self-exploration, without purpose or expectation. This non-striving attitude ensures that practitioners maintain awareness without a goal-oriented will or the tendency to over-control the body, allowing habitual or compensatory pathways to weaken. Second, while practicing, BMAA practitioners are taught to gently turn the gaze downward and inward—often synchronized with a slow exhalation—to quiet deliberative thoughts and return awareness to the moving body (for other psychological effects of this technique and state, see Ju and Lien, 2016; Teng and Lien, 2022; Wu et al., 2022). This distinguishes BMAA from mindfulness-based training in its handling of mental products; whereas the latter often involves a non-judgmental “mental observation” of thoughts and feelings, BMAA emphasizes diminishing these mental products by returning awareness to the somatic axis or movement itself. Third, insights on body–mind interaction derived from our long-term studies and feedback from hundreds of practitioners are also integrated. This integration helps practitioners comprehend and accept the emerging feelings and psychosomatic changes that arise during the course, which may be unfamiliar or even unsettling, particularly for those with a history of trauma.

In this way, the restored peaceful state, though brief and unstable at first, becomes more accessible as body fluidity increases. As the body becomes more unobstructed, suppressed emotions can be better accommodated and released as they gradually surface in a nonlinear process that advances at each person’s own pace. BMAA distinguishes itself from other mind–body practices in several key respects. To begin with, unlike other embodied therapeutic approaches, such as Somatic Experiencing (Levine, 1997, 2010), BMAA is a guided self-practice method that does not focus on symptoms, making it suitable for educational and preventive use in group settings. Additionally, BMAA uniquely defines an ideal body–mind state that serves as both an indicator and a direction for self-regulation, a role no other method explicitly addresses. Moreover, the method highlights the body’s agency by emphasizing movement with minimal mental interference. Finally, BMAA translates experiential philosophies that have long been implicit in East Asian traditions into an explicit, coherent set of principles for a structured psychological intervention. Unlike martial arts, which share the same roots, BMAA prioritizes inward-oriented self-regulation rather than outward-oriented objectives.

In summary, BMAA is a culturally rooted embodied approach to emotion regulation. Through enhanced body awareness, it deepens the connection to the body, thereby strengthening SoA and fostering dynamic self-regulation skills, as evidenced by the expected decrease in depressive tendencies. To test our hypothesis, we conducted a 10-week (30-h) intervention study with a pre-test and post-test design and an active control group.

## 2 Materials and methods

### 2.1 Participants

This study included participants who enrolled in a semester-long course titled “Mind-Body Axial Awareness and Mindfulness: Practice and Literature Review” at National Taiwan University. The course was offered in two successive sections on the same day. To mitigate selection bias, participants chose their preferred section time without prior knowledge of the group assignment (training or active-control). The necessary sample size of at least 62 participants was determined to detect a large effect size ( $\eta_{p}^{2}$ = 0.15), as reported by Teng and Lien (2016), with over 90% power (two-tailed). The power analysis was conducted for a between-participants design with a covariate using G*Power software (Faul et al., 2007).

Because of the COVID-19 pandemic’s restrictions on enrollments, we recruited two cohorts of participants. A total of 71 undergraduate and graduate students participated in the course during the spring and fall semesters of 2021. The initial group consisted of 50 students in the training group (28 from the first semester and 22 from the second semester) and 21 students in the active-control group (all from the first semester). Five participants missed the post-test, leaving a final sample of 66 for the analyses. The final sample included 47 participants in the training group (35 females; *Mean* age = 22.09 years, SD = 2.94) and 19 in the control group (10 females; *Mean* age = 22.62 years, SD = 4.42). A preliminary analysis confirmed that the two groups were comparable at baseline, showing no significant differences in age (*p* = 0.559) or gender (*p* = 0.085).

This study was approved by the Ministry of Education in Taiwan and was considered exempt from ethical review because it was conducted as part of a regular university course. A full description of the study was provided to all participants prior to enrollment, and written informed consent was obtained before the pre-test session.

### 2.2 Design and procedures

This study utilized a pretest-posttest design with an active control group. The intervention consisted of a 30-h BMAA or alternative program delivered over 10 consecutive weeks, structured as a required part of a semester-long university course. The training group received instruction and practice exclusively focused on the BMAA method, while the active control group engaged in literature discussions centered on embodied psychological themes. These discussions involved reviewing relevant contemplative practices (e.g., mindfulness-based or movement-based methods) that contrast with or are pertinent to BMAA practice, ensuring participants remained actively engaged in a similar mind–body issue for an equivalent duration.

Pre- and post-tests were administered one week before and one week after the intervention, respectively. During these measurement sessions, participants completed one-hour-long questionnaires measuring depression, sense of agency, and interoceptive sensibility, among other measures included for separate study purposes. The post-test marked the conclusion of data collection for this study. Afterwards, the groups switched learning activities for the rest of the semester to complete the course requirement.

**Figure 1:**
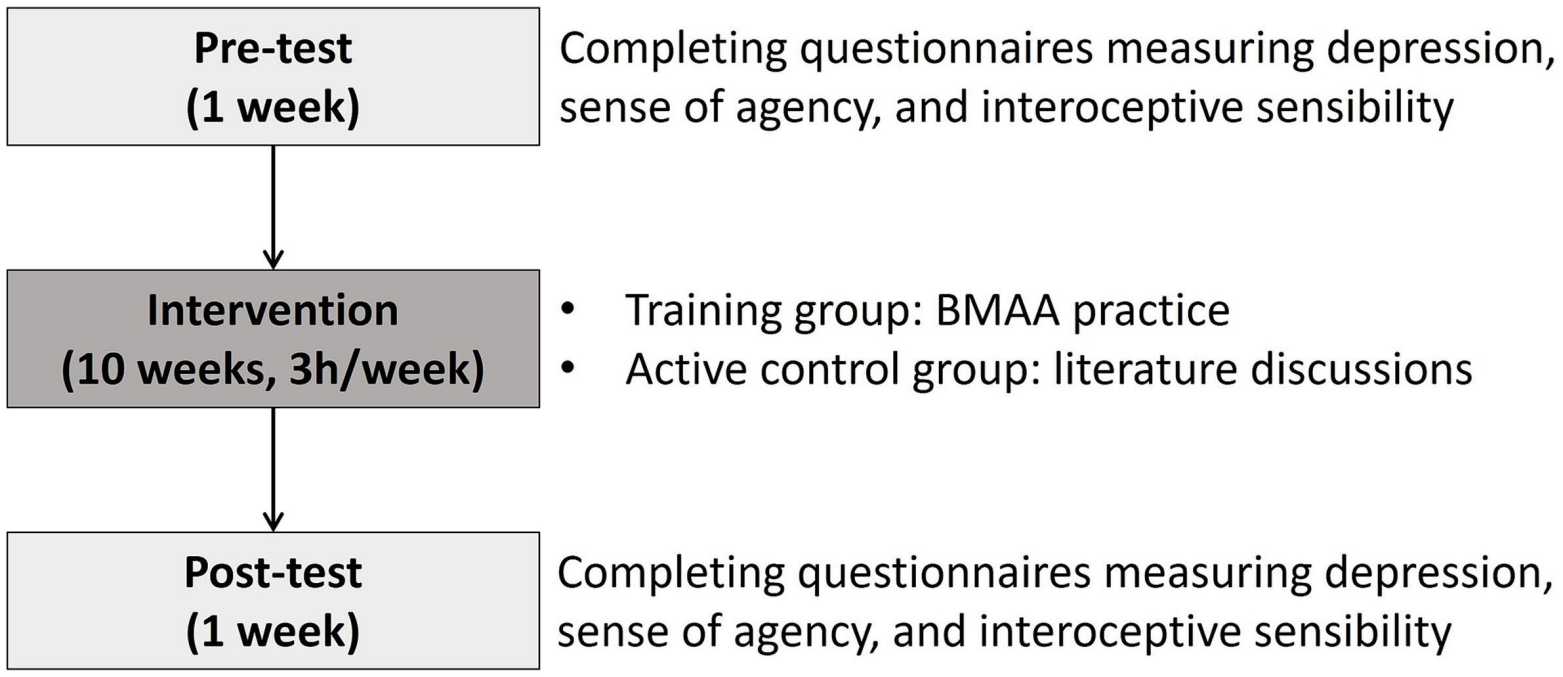
The study design and procedures.

To ensure consistency and minimize instructor effects, the same instructor (the first author) taught both the training and active control groups in the same physical classroom throughout the study. However, due to the COVID-19 pandemic outbreak, the delivery methods were modified during the final two weeks of the intervention for the first cohort: the training group received a blended format (a smaller in-person class combined with online sessions), while the control group participated entirely online. The design and procedures for both groups are shown in Figure 1.

#### 2.2.1 The intervention courses

The intervention consisted of two separate conditions: the BMAA training for the training group and a literature discussion course for the active control group.

Participants in the training group completed a 10-session BMAA program. Each session followed a structured tripartite format: (1) Somatic Practice (approximately 120 min), consisting of guided experiential exercises led by an instructor and a teaching assistant, incorporating both a review of prior practices and the introduction of new movements; (2) Psychological Guidance (approximately 30 min), involving the elucidation of core principles, concepts, and practice tips to facilitate deeper somatic understanding; and (3) Group Discussion (approximately 30 min), an instructor-led forum for participants to share subjective experiences, challenges, and reflections while receiving peer and instructor feedback.

To foster ongoing engagement and ecological application, two types of assignments were provided after each session. First, Reflective Journaling required participants to document their somatic feelings during practice and their subsequent phenomenological reflections in a secure online journal, with weekly brief feedback provided by the instructor. Second, the Daily Routine Application encouraged participants to integrate BMAA principles into their everyday lives. Rather than a rigid requirement, this involved finding opportunities to observe and adjust habitual patterns—such as breathing, walking, sitting, or using digital devices—by applying BMAA principles and techniques to maintain an axial state based on internal somatic feedback. An overview of the somatic practice, psychological guidance, and after-class assignments is provided in Supplementary Table 1.

The active-control group was designed to match the BMAA training group in terms of time commitment, social interaction, and cognitive engagement, while intentionally excluding BMAA’s specific embodied practices. Instead of hands-on practice, this group focused on active literature discussions related to embodied psychological themes. Each class session began with participants engaging in small-group discussions based on a pre-selected academic article (chosen from a curated list related to the weekly topic). This was followed by a whole-class sharing session where the instructor facilitated, integrated, and elaborated on the discussed literature and core concepts. To mirror the training group’s required out-of-class engagement, the active-control participants were also assigned preparatory tasks. They were asked to preview an article of their choice from the weekly topic list and record their questions, reflections, or critiques about it in a shared online journal before the upcoming class discussion.

### 2.3 Materials and tasks

#### 2.3.1 Chinese version of the Beck depression inventory-II (C-BDI-II)

To measure participants’ depressive symptoms, we used the Chinese version of the Beck Depression Inventory-II (BDI-II), translated by Chen (2000) from its original form by Beck et al. (1996). The C-BDI-II is a 21-item, 4-point (0–3) Likert-type scale that has consistently shown strong reliability, including a split-half reliability of 0.91 and an internal consistency of 0.94 (Cronbach’s *α*) in prior research (Lu et al., 2002). The internal consistency in our sample remained high at 0.93. The total score, ranging from 0 to 63, was calculated by summing the 21 items, with higher scores indicating greater depression severity. Scores were categorized as minimal (0–9), mild (10–18), moderate (19–29), and severe depression (30–63).

#### 2.3.2 Multidimensional assessment of interoceptive awareness-II (MAIA-II)

MAIA-II (Mehling et al., 2018) was used to measure participants’ interoceptive sensibility across multiple dimensions. The instrument is a 37-item, 6-point (0–5) Likert-type scale with eight subscales: (1) Noticing: an awareness of body sensations, including uncomfortable, comfortable, and neutral ones; (2) Not-Distracting: the ability to remain present with uncomfortable sensations; (3) Not-Worrying: the tendency not to experience emotional distress in response to discomfort; (4) Attention Regulation: the ability to sustain and control attention on body sensations; (5) Emotional Awareness: the awareness of the connection between body sensations and emotional states; (6) Self-Regulation: the ability to regulate distress by attending to body sensations; (7) Body Listening: actively listening to the body for insight; and (8) Trusting: a sense of one’s body as safe and trustworthy. The Cronbach’s alphas for these subscales ranged from 0.64 to 0.83 in Mehling et al. (2018), while in our sample, they ranged from 0.50 to 0.83. Mean scores for each subscale, ranging from 0 to 5, were calculated as indicators of various dimensions of interoceptive sensibility, with higher scores reflecting a greater sensibility.

#### 2.3.3 Sense of agency scale (SoAS)

Participants’ sense of agency was measured using the SoAS (Tapal et al., 2017). The scale is an 11-item, 7-point (1–7) Likert-type scale consisting of two subscales: Sense of Positive Agency (SoPA), which is the feeling of being in control of one’s body, mind, and environment; and Sense of Negative Agency (SoNA), the feeling that these factors are not under one’s control. Both subscales have shown good internal consistency, with the original study reporting a McDonald’s *ω* of 0.78 for SoPA and 0.76 for SoNA. In our sample, the internal consistency (Cronbach’s *α*) for the two subscales was 0.75 and 0.74, respectively. The mean scores for each subscale were calculated to represent different facets of agency, ranging from 1 to 7. Higher scores on the respective subscales indicated a greater sense of positive or negative agency.

## 3 Results

In this section, we first analyzed the training effects of BMAA on depression (BDI scores), sense of agency (SoPA and SoNA scores), and interoceptive sensibility (MAIA subscale scores). Next, we investigated whether BMAA training alleviated depression by improving interoceptive sensibility and the sense of agency.

The pre- and post-test scores for all questionnaires are shown in Table 1. At baseline, there were no significant differences between the training and active-control groups in measures of the sense of agency and most dimensions of interoceptive sensibility (*p* > 0.10), except for the Not-Worrying dimension of MAIA. The independent t-test showed that the training group had significantly lower pre-test scores on this subscale (*M* = 1.36, SD = 0.65) compared to the control group (*M* = 1.73, SD = 0.67), *t*(64) = −2.04, *p* = 0.046.

**Table 1:** Mean (SD) scores and ANCOVA statistics for BDI, MAIA, and SoAS in the BMAA training and active-control groups.

Scale	Subscale	Pretest		Posttest		ANCOVA			
		Training group	Control group	Training group	Control group	*F*	MSE	*p*-value	$\eta_{P}^{2}$
BDI		15.62	11.00	7.89	11.95	8.41	51.52	0.005	0.12
		(11.11)	(6.76)	(7.15)	(10.02)				
MAIA	Noticing	3.11	3.30	3.87	3.32	18.01	0.28	<0.001	0.22
		(0.69)	(0.77)	(0.60)	(0.54)				
	Not-distracting	1.75	1.86	2.82	2.11	16.37	0.45	<0.001	0.21
		(0.71)	(0.66)	(0.73)	(0.60)				
	Not-worrying	1.36	1.73	2.30	1.80	12.41	0.45	0.001	0.17
		(0.65)	(0.67)	(0.75)	(0.64)				
	Attention regulation	2.31	2.64	3.38	2.85	17.77	0.34	<0.001	0.22
		(0.74)	(0.90)	(0.67)	(0.70)				
	Emotional awareness	3.30	3.38	3.92	3.43	16.79	0.21	<0.001	0.21
		(0.69)	(0.67)	(0.49)	(0.58)				
	Self-regulation	2.65	2.88	3.66	3.02	20.98	0.34	<0.001	0.25
		(0.73)	(0.87)	(0.61)	(0.77)				
	Body listening	2.68	2.84	3.56	2.89	17.54	0.40	<0.001	0.22
		(0.89)	(0.82)	(0.71)	(0.69)				
	Trusting	2.88	3.25	3.62	3.22	6.07	0.67	0.016	0.09
		(1.04)	(0.95)	(0.93)	(0.85)				
SoAS	SoPA	4.69	4.84	5.04	4.91	1.36	0.44	0.249	0.02
		(0.98)	(0.86)	(0.81)	(0.87)				
	SoNA	2.96	2.55	2.40	2.66	6.61	0.41	0.013	0.10
		(0.97)	(0.78)	(0.75)	(0.86)				

However, the training group scored significantly higher on the BDI (possible range: 1–41, *M* = 15.62, SD = 11.11) than the control group (possible range: 0–26, *M* = 11.00, SD = 6.76), *t*(53.71) = 2.06, *p* = 0.044. Notably, the proportion of participants in the training group experiencing moderate or severe depression (31.9%) was higher than in the control group (10.5%), with no participants in the control group experiencing severe depression.

Due to baseline differences in BDI and the MAIA Not-Worrying subscale, a series of one-way ANCOVAs was performed to assess the training effects of BMAA while statistically controlling for these initial group differences.

### 3.1 BMAA training effects on depressive tendencies

The ANCOVA analysis showed that, after controlling for pre-test scores, the training group had significantly lower post-test scores on the BDI (Depression) compared to the control group (*M* = 7.89 vs. 11.95), *F*(1, 63) = 8.41, *MSE* = 51.52, *p* = 0.005, $\eta_{P}^{2}$ ​ = 0.12. This result indicates that the BMAA training group experienced a significantly greater reduction in depressive symptoms than the control group. This effect remained significant even after excluding participants with severe pre-test depression (*M* = 6.56 vs. 11.95, *F*(1, 55) = 8.27, MSE = 49.22, *p* = 0.006, $\eta_{P}^{2}$ = 0.13), suggesting that the reduction in depression was robust and not solely driven by those with the most potential for improvement.

### 3.2 BMAA training effects on interoceptive sensibility

As shown in Table 1, the ANCOVA analyses revealed that the training group achieved significantly higher post-test scores on all eight MAIA subscales compared to the control group (all *p* ≤ 0.02; all $\eta_{P}^{2}$ ≥ 0.08). This comprehensive pattern of results indicates that BMAA training significantly enhances interoceptive sensibility across all dimensions. Specifically, participants demonstrated a greater tendency to notice and maintain attention to body sensations, avoid ignoring or worrying about discomfort, regulate distress by focusing on body sensations, recognize the connection between body sensations and emotional states, and perceive their bodies as safe and trustworthy.

### 3.3 BMAA training effects on sense of agency

The ANCOVA analyses revealed a significant group effect on the SoNA, with the training group having a lower mean score ( M = 2.40) compared to the control group ( M = 2.66), F (1, 63) = 6.61, MSE = 0.41, p = 0.013, $\eta_{P}^{2}$ = 0.10. This result showed that, after the BMAA training, participants experienced significantly fewer feelings that their body, mind, and environment were out of their control compared to the control group, indicating a beneficial reduction in the negative narrative of agency. In contrast, there was no significant effect on the SoPA (training group: *M* = 5.04 vs. control group: *M* = 4.91), *F*(1, 63) = 1.36, MSE = 0.44, *p* = 0.249, $\eta_{P}^{2}$ = 0.02.

### 3.4 Correlation of change in variables

To explore the potential paths involving interoceptive sensibility and SoA that may underlie the positive effects of BMAA training, Pearson correlation coefficients were calculated between the pretest-to-posttest change scores (Δ) for all variables in all participants. The results are shown in Table 2.

**Table 2:** Correlation coefficients for changes in all variables.

No.	Variables	2	3	4	5	6	7	8	9	10	11
1	BDI	−0.19	0.49^***^	−0.07	−0.30^*^	−0.37^**^	−0.38^**^	−0.16	−0.40^**^	−0.17	−0.30^*^
2	SoPA	–	−0.32^**^	−0.01	0.07	0.27^*^	0.21^†^	−0.06	0.05	0.08	−0.02
3	SoNA		–	−0.19	−0.46^***^	−0.2	−0.40^**^	−0.1	−0.36^**^	−0.26^*^	−0.36^**^
4	Noticing			–	0.28^*^	0.17	0.56^***^	0.64^***^	0.35^**^	0.41^**^	0.50^***^
5	Not-distracting				–	0.23^†^	0.56^***^	0.37^**^	0.43^***^	0.43^***^	0.35^**^
6	Not-worrying					–	0.45^***^	0.29^*^	0.27^*^	0.26^*^	0.28^*^
7	Attention regulation						–	0.47^***^	0.64^***^	0.52^***^	0.56^***^
8	Emotional awareness							–	0.38^**^	0.46^***^	0.47^***^
9	Self-regulation								–	0.54^***^	0.53^***^
10	Body listening									–	0.52^***^
11	Trusting										–

^†^0.05 ≤ *p* < 0.10, ^*^p < 0.05, ^**^p < 0.01, ^***^p < 0.001.

#### 3.4.1 Correlates of change in depression

As shown, the decrease in depressive symptoms (ΔBDI) significantly correlates with changes in several key variables. First, it was positively correlated with ΔSoNA, *r*(66) = 0.49, *p* < 0.001. Since a reduction in both BDI (less depression) and SoNA (less negative sense of agency) is an adverse change in score, this positive correlation indicates that improvement in depressive symptoms is strongly linked to a decrease in participants’ feelings of failure or lack of control over their lives and actions. This also suggests a potential positive therapeutic connection. However, no significant relationship was found between ΔBDI and ΔSoPA, *r*(66) = −0.19, *p* = 0.127.

Second, ΔBDI was negatively correlated with increases in five MAIA dimensions: Not-Distracting (*r*(66) = −0.31, *p* = 0.013); Not-Worrying (*r*(66) = −0.38, *p* = 0.002); Attention Regulation (*r*(66) = −0.38, *p* = 0.002); Self-Regulation (*r*(66) = −0.40, *p* = 0.001); and Trusting (*r*(66) = −0.30, *p* = 0.014). No correlation was found for the other three subscales (all *p* > 0.10). This indicates that as depressive symptoms decrease, participants show greater ability to stay with and manage bodily sensations without distraction or worry, to regulate themselves, to pay attention, and to trust their body signals.

#### 3.4.2 Correlation between change in SoA and interoceptive sensibility

The ΔSoNA was significantly negatively correlated with increases in five MAIA dimensions: Not-Distracting (*r*(66) = −0.46, *p* < 0.001), Attention Regulation (*r*(66) = −0.40, *p* = 0.001), Self-Regulation (*r*(66) = −0.36, *p* = 0.003), Body Listening (*r*(66) = −0.26, *p* = 0.032), and Trusting (*r*(66) = −0.36, *p* = 0.003). These strong negative correlations suggest that a decrease in the negative sense of agency is closely linked to a more accepting, manageable, and trusting interoceptive relationship. In contrast, only the improvement in the Not-Worrying dimension of MAIA was significantly positively correlated with ΔSoPA, *r*(66) = 0.27, *p* = 0.028. The other subscales did not reach significance (all *p* > 0.09). Unlike SoNA, this indicates that an increase in a positive narrative of SoA is only associated with a better ability to avoid worry about bodily sensations.

### 3.5 Mechanisms of change in depression: serial mediation analyses

To investigate the hypothesized mechanism underlying the reduction effect of the BMAA training on depression, eight serial multiple mediation models were tested. Each model involved one of the eight interoceptive sensibility subscales and the SoNA to determine whether they sequentially mediate the positive effect of BMAA training on depressive tendencies. The change in SoPA was excluded from these models because of a nonsignificant training effect. The hypothesized mechanism suggests an indirect pathway, where BMAA Group status initially improves interoception, which then lessens the negative sense of agency, leading to a decrease in depression (Group → ΔMAIA → ΔSoNA → ΔBDI). The eight specific models examined are listed in Table 3.

**Table 3:** The independent variable, dependent variable, and serial mediators used in the eight serial multiple mediation models.

Model	Independent variable	Serial mediators		Dependent variable
		M1	M2	
Model 1	Group	Noticing	SoNA	Depression (BDI)
Model 2	Group	Not-distracting	SoNA	Depression (BDI)
Model 3	Group	Not-worrying	SoNA	Depression (BDI)
Model 4	Group	Attention regulation	SoNA	Depression (BDI)
Model 5	Group	Emotional awareness	SoNA	Depression (BDI)
Model 6	Group	Self-regulation	SoNA	Depression (BDI)
Model 7	Group	Body listening	SoNA	Depression (BDI)
Model 8	Group	Trusting	SoNA	Depression (BDI)

**Figure 2:**
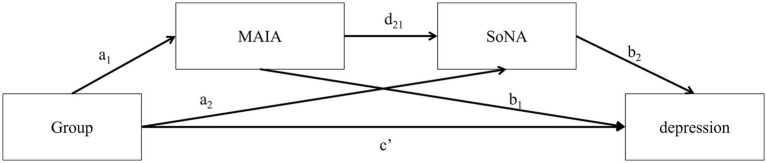
Hypothesized serial multiple mediation model examining the effect of BMAA training on depression through MAIA subscales and SoNA. Arrows indicate hypothesized directional relationships.

As shown in Figure 2, these models used Group (BMAA training vs. Control) as the independent variable, the change scores of BDI as the dependent variable, and the change scores of one of the eight MAIA subscales and SoNA as the serial mediators. All analyses were conducted using the PROCESS macro for R (version 4.3.1; Hayes, 2022). A bias-corrected bootstrap method, with 10,000 iterations and a 95% Confidence Interval (CI), was employed to evaluate significant indirect effects and path coefficients.

#### 3.5.1 Test of serial mediation (model 1): BMAA training effects on depression via ΔNoticing and ΔSoNA

The results for Model 1, testing the serial path through the Noticing subscale of interoceptive sensibility and SoNA, indicated that the hypothesized serial indirect effect was not statistically significant, *B* = −0.19, *SE* = 0.52, 95% CI = [−1.37, 0.81]. The indirect effect of the path through the Noticing subscale alone was not significant, either (*B* = 1.24, *SE* = 1.29, 95% CI = [−1.23, 3.88]).

**Figure 3:**
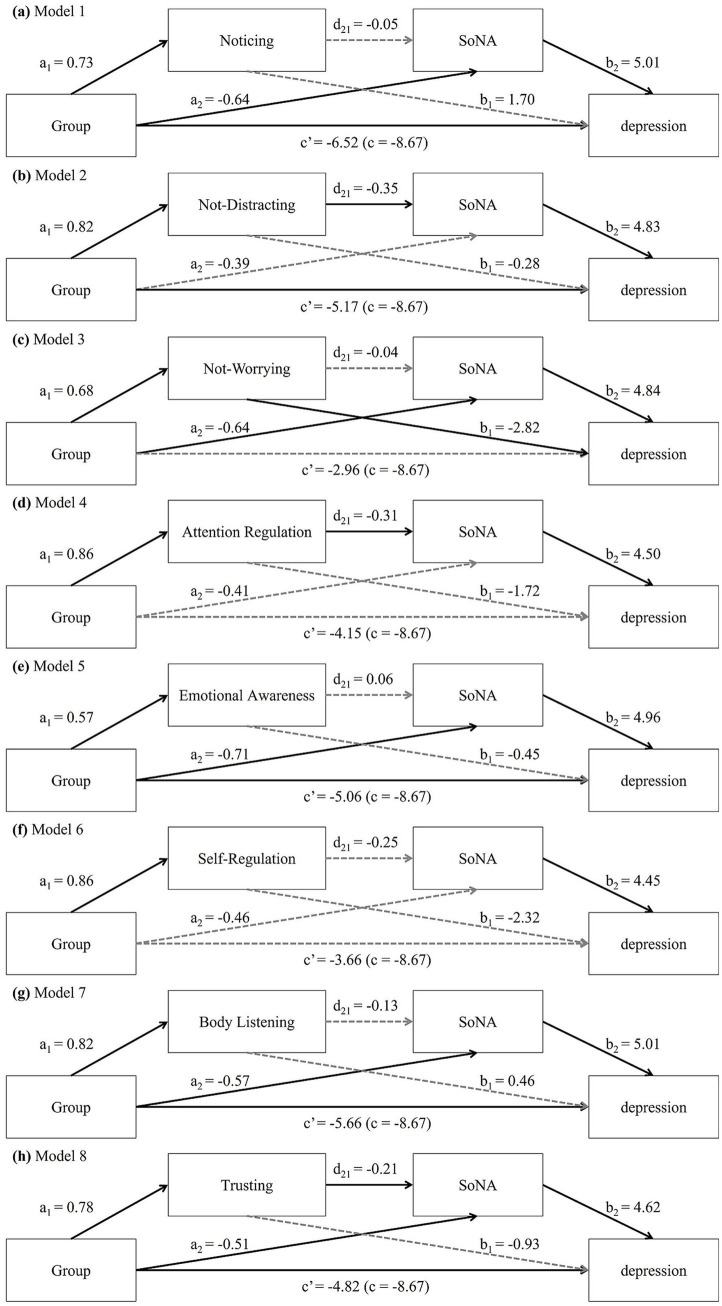
Serial multiple mediation model for the effect of group on depression (measured with BDI) through each subscale of interoceptive sensibility (measured with MAIA) and sense of negative agency (SoNA; measured with sense of agency scale). Each panel represents a separate model including one MAIA subscale as the first mediator: **(a)** Noticing, **(b)** Not-Distracting, **(c)** Not-Worrying, **(d)** Attention Regulation, **(e)** Emotional Awareness, **(f)** Self-Regulation, **(g)** Body Listening, and **(h)** Trusting. Solid lines indicate significant paths; dashed lines indicate non-significant paths.

However, the indirect effect of the path through SoNA alone was significant, *B* = −3.20, SE = 1.77, 95% CI = [−7.25, −0.49]. As detailed in Table 4, this path was influenced by the BMAA training group, significantly predicting a reduction in SoNA (*a*_2_ = −0.64, SE = 0.24, 95% CI = [−1.13, −0.17]), which then significantly predicted a decrease in BDI (*b*_2_ = 5.01, SE = 1.64, 95% CI = [1.73, 8.16]). These results suggest that the BMAA training could reduce depressive tendencies by lowering feelings of failure and lack of control. Additionally, the direct effect of Group on BDI remained substantial after including these serial and single mediators (*c*′ = −6.52, SE = 2.61, 95% CI = [−11.69, −1.46]), indicating a partial serial mediating effect. Figure 3a displays Model 1 and presents the estimates of the path coefficients.

**Table 4:** Regression coefficients, standard errors, and confidence intervals for the eight serial multiple mediation models

Model	Antecedent	Path	M1			Path	M2 (SoNA)			Path	Y (depression)		
			Coeff.	Boot. SE	Boot. 95% CI		Coeff.	Boot. SE	Boot. 95% CI		Coeff.	Boot. SE	Boot. 95% CI
Model 1	X (group)	*a*_1_	0.73	0.17	[0.41, 1.06]	*a*_2_	−0.64	0.24	[−1.13, −0.17]	*c*′	−6.52	2.61	[−11.69, −1.46]
	M1 (Noticing)					*d*_21_	−0.05	0.13	[−0.29, 0.22]	*b*_1_	1.70	1.74	[−1.64, 5.26]
	M2 (SoNA)									*b*_2_	5.01	1.64	[1.73, 8.16]
	Constant		0.03	0.13	[−0.22, 0.27]		0.12	0.18	[−0.24, 0.49]		0.33	1.59	[−2.79, 3.57]
		*F*(1, 64) = 14.69, *p* < 0.001 *R*^2^ = 0.19				*F*(2, 63) = 4.89, *p* = 0.011 *R*^2^ = 0.13				*F*(3, 62) = 9.20, *p* < 0.001 *R*^2^ = 0.31			
Model 2	X (group)	*a*_1_	0.26	0.12	[0.02, 0.50]	*a*_2_	−0.39	0.23	[−0.86, 0.03]	*c*′	−5.17	2.36	[−9.75, −0.51]
	M1 (Not-distracting)					*d*_21_	−0.35	0.11	[−0.55, −0.13]	*b*_1_	−0.28	1.49	[−3.25, 2.58]
	M2 (SoNA)									*b*_2_	4.83	1.73	[1.29, 8.01]
	Constant		0.82	0.18	[0.46, 1.17]		0.20	0.18	[−0.14, 0.57]		0.47	1.57	[−2.56, 3.61]
		*F*(1, 64) = 12.91, *p* < 0.001 *R*^2^ = 0.17				*F*(2, 63) = 10.35, *p* < 0.001 *R*^2^ = 0.25				*F*(3, 62) = 8.66, *p* < 0.001 *R*^2^ = 0.30			
Model 3	X (group)	*a*_1_	0.86	0.18	[0.52, 1.22]	*a*_2_	−0.64	0.21	[−1.06, −0.24]	*c*′	−2.96	2.55	[−8.06, 1.94]
	M1 (Not-worrying)					*d*_21_	−0.04	0.13	[−0.31, 0.21]	*b*_1_	−2.82	1.42	[−5.66, −0.01]
	M2 (SoNA)									*b*_2_	4.84	1.49	[1.82, 7.68]
	Constant		0.07	0.13	[−0.20, 0.34]		0.12	0.18	[−0.24, 0.48]		0.60	1.54	[−2.35, 3.67]
		*F*(1, 64) = 17.57, *p* < 0.001 *R*^2^ = 0.22				*F*(2, 63) = 4.86, *p* = 0.011 *R*^2^ = 0.13				*F*(3, 62) = 10.53, *p* < 0.001 *R*^2^ = 0.34			
Model 4	X (group)	*a*_1_	0.86	0.17	[0.52, 1.18]	*a*_2_	4.50	0.21	[−0.80, 0.01]	*c*′	−4.15	2.37	[−8.88, 0.36]
	M1 (Attention regulation)					*d*_21_	−0.31	0.12	[−0.56, −0.09]	*b*_1_	−1.72	1.25	[−4.12, 0.85]
	M2 (SoNA)									*b*_2_	450	1.63	[1.23, 7.64]
	Constant		0.21	0.12	[−0.02, 0.47]		0.18	0.18	[−0.18, 0.55]		0.81	1.59	[−2.25, 4.00]
		*F*(1, 64) = 12.24, *p* < 0.001 *R*^2^ = 0.23				*F*(2, 63) = 8.01, *p* < 0.001 *R*^2^ = 0.21				*F*(3, 62) = 9.20, *p* < 0.001 *R*^2^ = 0.31			
Model 5	X (group)	*a*_1_	0.57	0.14	[0.30, 0.84]	*a*_2_	−0.71	0.23	[−1.16, −0.26]	*c*′	−5.06	2.44	[−9.93, −0.40]
	M1 (Emotional awareness)					*d*_21_	0.06	0.16	[−0.27, 0.35]	*b*_1_	−0.45	1.83	[−4.12, 3.08]
	M2 (SoNA)									*b*_2_	4.96	1.67	[1.47, 8.05]
	Constant		0.05	0.09	[−0.12, 0.23]		0.11	0.18	[−0.23, 0.48]		0.41	1.54	[−2.55, 3.50]
		*F*(1, 64) = 10.77, *p* = 0.002 *R*^2^ = 0.14				*F*(2, 63) = 4.89, *p* = 0.011 *R*^2^ = 0.13				*F*(3, 62) = 8.67, *p* < 0.001 *R*^2^ = 0.30			
Model 6	X (group)	*a*_1`_	0.86	0.21	[0.44, 1.28]	*a*_2_	−0.46	0.23	[−0.88, 0.02]	*c*′	−3.66	2.46	[−8.49, 1.08]
	M1 (Self-regulation)					*d*_21_	−0.25	0.16	[−0.55, 0.06]	*b*_1_	−2.32	1.46	[−5.07, 0.68]
	M2 (SoNA)									*b*_2_	4.45	1.49	[1.42, 7.25]
	Constant		0.14	0.19	[−0.23, 0.51]		0.15	0.20	[−0.23, 0.53]		0.77	1.47	[−2.21, 3.62]
		*F*(1, 64) = 18.92, *p* < 0.001 *R*^2^ = 0.23				*F*(2, 63) = 6.86, *p* = 0.002 *R*^2^ = 0.18				*F*(3, 62) = 9.73, *p* < 0.001 *R*^2^ = 0.32			
Model 7	X (group)	*a*_1_	0.82	0.21	[0.42, 1.23]	*a*_2_	−0.57	0.22	[−1.00, −0.14]	*c*′	−5.66	2.63	[−10.72, −0.37]
	M1 (Body listening)					*d*_21_	−0.13	0.13	[−0.41, 0.12]	*b*_1_	0.46	1.48	[−2.45, 3.35]
	M2 (SoNA)									*b*_2_	5.01	1.61	[1.83, 8.15]
	constant		0.06	0.16	[−0.26, 0.37]		0.12	0.19	[−0.24, 0.52]		0.35	1.56	[−2.67, 3.49]
		*F*(1, 64) = 12.90, *p* = 0.001 *R*^2^ = 0.17				*F*(2, 63) = 5.53, *p* = 0.006 *R*^2^ = 0.15				*F*(3, 62) = 8.69, *p* < 0.001 *R*^2^ = 0.30			
Model 8	X (group)	*a*_1_	0.78	0.26	[0.26, 1.28]	*a*_2_	−0.51	0.21	[−0.91, −0.09]	*c*′	−4.82	2.36	[−9.39, −0.18]
	M1 (Trusting)					*d*_21_	−0.21	0.09	[−0.40, −0.04]	*b*_1_	−0.93	1.14	[−3.13, 1.43]
	M2 (SoNA)									*b*_2_	4.62	1.54	[1.48, 7.59]
	Constant		−0.04	0.21	[−0.44, 0.40]		0.11	0.18	[−0.26, 0.47]		0.38	1.52	[−2.66, 3.38]
		*F*(1, 64) = 8.07, *p* = 0.006 *R*^2^ = 0.11				*F*(2, 63) = 7.61, *p* = 0.001 *R*^2^ = 0.19				*F*(3, 62) = 8.96, *p* < 0.001 *R*^2^ = 0.30			

#### 3.5.2 Test of serial mediation (model 2): BMAA training effects on depression via ΔNot-distracting and ΔSoNA

The results for Model 2, testing the serial path through the Not-Distracting subscale of MAIA and SoNA, confirmed that the hypothesized indirect effect was statistically significant, *B* = −0.14, SE = 0.63, 95% CI = [−2.70, −0.26].

As shown in Table 4, all the individual path coefficients contributing to this indirect effect were significant and aligned with the proposed mechanism. First, the BMAA training group significantly predicted an increase in the Not-Distracting subscale (*a*_1_ = 0.82, SE = 0.18, 95% CI = [0.46, 1.17]). Second, this increase then significantly predicted a reduction in SoNA (*d*_12_ = −0.35, SE = 0.11, 95% CI = [−0.55, −0.13]). Finally, the decrease in SoNA significantly predicted a reduction in BDI (*b*_2_ = 4.83, SE = 1.73, 95% CI = [1.29, 8.01]). This significant serial mediation path indicates that BMAA training reduces depressive tendencies by initially enhancing practitioners’ ability to stay present with uncomfortable bodily sensations, which subsequently leads to a decrease in feelings of failure and lack of control.

The other two non-serial indirect effects (through the Not-Distracting subscale alone or SoNA alone) were not significant (Not-Distracting alone: *B* = −0.23, SE = 1.25, 95% CI = [−2.92, 2.10]; SoNA alone: *B* = −1.90, SE = 1.51, 95% CI = [−5.55, 0.12]). Additionally, the direct effect of Group on BDI remained substantial after including these serial mediators (*c*′ = −5.17, SE = 2.36, 95% CI = [−9.75, −0.51]), indicating a partial serial mediating effect. Figure 3b displays Model 2 and presents the estimates of the path coefficients.

#### 3.5.3 Test of serial mediation (model 3): BMAA training effects on depression via ΔNot-worrying and ΔSoNA

The results for Model 3, testing the serial path through the Not-Worrying subscale of interoceptive sensibility and SoNA, indicated that the hypothesized serial indirect effect was not statistically significant, *B* = −0.02, SE = 0.06, 95% CI = [−0.16, 0.08].

However, two independent indirect effects were statistically significant: the path through the Not-Worrying subscale alone (*B* = −0.24, SE = 0.14, 95% CI = [−0.53, −0.00]) and the path through SoNA alone (*B* = −0.30, SE = 0.12, 95% CI = [−0.56, −0.08]). As detailed in Table 4, the first path was significantly influenced by the BMAA training group, which then predicted an increase in the Not-Worrying subscale (*a*_1_ = 0.86, SE = 0.18, 95% CI = [0.52, 1.22]). This increase then significantly predicted a decrease in BDI (*b*_1_ = −2.82, SE = 1.42, 95% CI = [−5.66, −0.01]). The second path was influenced by the BMAA training group, significantly predicting a reduction in SoNA (*a*_2_ = −0.64, SE = 0.21, 95% CI = [−1.06, −0.24]), which then significantly predicted a decrease in BDI (*b*_2_ = 4.84, SE = 1.49, 95% CI = [1.82, 7.68]). These results suggest that the BMAA training could reduce depressive tendencies either by increasing the tendency not to worry about discomfort or by lowering the negative sense of agency. Additionally, the direct effect of Group on BDI was no longer significant after including the mediators (*c*′ = −2.96, SE = 2.55, 95% CI = [−8.06, 1.94]), indicating full mediation. Figure 3c illustrates Model 3 and shows the path coefficient estimates.

#### 3.5.4 Test of serial mediation (model 4): BMAA training effects on depression via ΔAttention regulation and ΔSoNA

The results for Model 4, testing the serial path through the Attention Regulation subscale and SoNA, confirmed a statistically significant indirect effect, *B* = −1.21, SE = 0.79, 95% CI = [−3.16, −0.12].

As shown in Table 4, all the individual path coefficients contributing to this indirect effect were significant and in the anticipated beneficial direction. First, the BMAA training group initially predicted an increase in the Attention Regulation dimension of interoceptive sensibility (*a*_1_ = 0.86, SE = 0.17, 95% CI = [0.52, 1.18]). Second, this enhanced Attention Regulation then significantly predicted a decrease in SoNA (*d*_12_ = −0.31, SE = 0.12, 95% CI = [−0.56, −0.09]). Finally, the decrease in SoNA significantly predicted the reduction in BDI (*b*_2_ = 4.50, SE = 1.63, 95% CI = [1.23, 7.64]). This significant serial path suggests that the BMAA training reduces depressive tendencies by first enhancing practitioners’ ability to sustain attention to their body sensations, which sequentially leads to a reduction in their sense of failure and lack of control.

The other two non-serial indirect effects (through Attention Regulation alone, or SoNA alone) were not significant (Attention Regulation alone: *B* = −1.48, SE = 1.08, 95% CI = [−3.59, 0.73]; SoNA alone: *B* = −1.83, SE = 1.26, 95% CI = [−4.81, 0.07]). Additionally, the direct effect of Group on BDI was no longer significant after the serial mediators were included (*c*′ = −4.15, SE = 2.37, 95% CI = [−8.88, 0.36]). This finding indicates a complete serial mediating effect for this specific path. Figure 3d illustrates Model 4 and shows the estimates of the path coefficients.

#### 3.5.5 Test of serial mediation (model 5): BMAA training effects on depression via ΔEmotional awareness and ΔSoNA

The results for Model 5, testing the serial path through the Emotional Awareness subscale of interoceptive sensibility and SoNA, indicated that the hypothesized serial indirect effect was not statistically significant, *B* = −0.16, SE = 0.45, 95% CI = [−0.86, 1.00]. The indirect effect of the path through the Emotional Awareness subscale alone was not significant, either (*B* = −0.26, SE = 1.08, 95% CI = [−2.46, 1.96]).

However, the indirect effect of the path through SoNA alone was significant, *B* = −3.51, SE = 1.77, 95% CI = [−7.46, −0.62]. As detailed in Table 4, this path was influenced by the BMAA training group, significantly predicting a reduction in SoNA (*a*_2_ = −0.71, SE = 0.23, 95% CI = [−1.16, −0.26]), which then significantly predicted a decrease in BDI (*b*_2_ = 4.96, SE = 1.67, 95% CI = [1.47, 8.05]). These results suggest that the BMAA training could reduce depressive tendencies by lowering the negative sense of agency. Additionally, the direct effect of Group on BDI remained substantial after including these serial and single mediators (*c*′ = −5.06, SE = 2.44, 95% CI = [−9.93, −0.40]), indicating a partial serial mediating effect. Figure 3e displays Model 5 and presents the estimates of the path coefficients.

#### 3.5.6 Test of serial mediation (model 6): BMAA training effects on depression via ΔSelf-regulation and ΔSoNA

The results for Model 3, testing the serial path through the Self-Regulation subscale and SoNA, showed that the hypothesized serial indirect effect was not statistically significant, *B* = −0.97, SE = 0.87, 95% CI = [−3.17, 0.19]. Furthermore, the two non-serial indirect effects (through Self-Regulation alone or SoNA alone) also failed to reach significance (Self-Regulation alone: *B* = −2.00, SE = 1.48, 95% CI = [−5.34, 0.54]; SoNA alone: *B* = −2.04, SE = 1.30, 95% CI = [−4.88, 0.08]). The direct effect of Group on BDI was no longer significant after the serial mediators were included (*c*′ = −3.66, SE = 2.46, 95% CI = [−8.49, 1.08]). Figure 3f displays Model 6 and presents the estimates of the path coefficients.

#### 3.5.7 Test of serial mediation (model 7): BMAA training effects on depression via ΔBody listening and ΔSoNA

The results for Model 7, testing the serial path through the Body Listening subscale of interoceptive sensibility and SoNA, indicated that the hypothesized serial indirect effect was not statistically significant, *B* = −0.54, SE = 0.60, 95% CI = [−1.86, 0.59]. The indirect effect of the path through the Body Listening subscale alone was not significant, either (*B* = 0.38, SE = 1.25, 95% CI = [−2.21, 2.84]).

However, the indirect effect of the path through SoNA alone was significant, *B* = −2.85, SE = 1.66, 95% CI = [−6.76, −0.38]. As detailed in Table 4, this path was influenced by the BMAA training group, significantly predicting a reduction in SoNA (*a*_2_ = −0.57, SE = 0.22, 95% CI = [−1.00, −0.14]), which then significantly predicted a decrease in BDI (*b*_2_ = 5.01, SE = 1.61, 95% CI = [1.83, 8.15]). These results suggest that the BMAA training could reduce depressive tendencies by lowering the sense of agency. Additionally, the direct effect of Group on BDI remained substantial after including these serial and single mediators (*c*′ = −5.66, SE = 2.63, 95% CI = [−10.72, −0.37]), indicating a partial serial mediating effect. Figure 3g displays Model 7 and presents the estimates of the path coefficients.

#### 3.5.8 Test of serial mediation (model 8): BMAA training effects on depression via ΔTrusting and ΔSoNA

The results of Model 8, which tested the serial path through the Trusting subscale of interoceptive sensibility and SoNA, confirmed that the hypothesized serial indirect effect was statistically significant, *B* = −0.76, SE = 0.59, 95% CI = [−2.28, −0.04].

As detailed in Table 4, all the individual path coefficients contributing to this serial path were significant and in a positive direction. First, the BMAA training group significantly predicted an increase in the Trusting subscale (*a*_1_ = 0.78, SE = 0.26, 95% CI = [0.26, 1.28]). Second, this increase then significantly predicted a decrease in SoNA (*d*_12_ = −0.21, SE = 0.09, 95% CI = [−0.40, −0.04]). Finally, the decrease in SoNA significantly predicted a reduction in BDI (*b*_2_ = 4.62, SE = 1.54, 95% CI = [1.48, 7.59]). This serial path indicates that the BMAA training lowers depressive tendencies by first increasing practitioners’ trust in their bodies as a safe and trustworthy place, which then sequentially reduces their feelings of failure and lack of control.

Additionally, the indirect effect through the path of SoNA alone was also significant (*B* = −2.37, SE = 1.34, 95% CI = [−5.39, −0.21]). This path was significantly influenced by the BMAA training group, which then predicted a reduction in SoNA (*a*_2_ = −0.51, SE = 0.21, 95% CI = [−0.91, −0.09]). In contrast, the indirect effect through the Trusting subscale alone was not significant (*B* = −0.72, SE = 1.04, 95% CI = [−3.12, 1.11]). The significance of the SoNA-only path suggests that the BMAA training could reduce depressive tendencies by directly impacting and lowering the negative sense of agency through pathways not fully captured by the Trusting subscale. Furthermore, the direct effect remained significant after including the serial and single mediators (*c*′ = −4.82, SE = 2.36, 95% CI = [−9.39, −0.18]), indicating a partial mediating effect overall. Figure 3h displays Model 8 and shows the path coefficient estimates.

## 4 Discussion

The present study aimed to examine the impact of a 10-week Body–Mind-Axial-Awareness (BMAA) practice on university students’ depression, interoceptive sensibility, and the sense of agency (SoA). We also investigated the sequential mediating roles of interoception and SoA in explaining the positive effects of BMAA on depressive tendencies. The main findings are summarized below.

First, as predicted, compared to the active control group, BMAA practice significantly lowered participants’ depressive tendencies (as measured by the BDI-II). It also enhanced all eight dimensions of interoceptive sensibility (as measured by the MAIA) and reduced negative sense of agency (SoNA) (as measured by the SoAS). Second, we demonstrated a sequential mediation pathway in which improvements in interoceptive sensibility indirectly reduced depressive tendencies via SoNA. Specifically, three MAIA dimensions—maintaining attention on body sensations (Attention Regulation), staying present with uncomfortable sensations (Not-Distracting), and developing a sense of the body as safe and trusting (Trusting)—each independently contributed to a decrease in SoNA, which then led to improved depression. Third, the reduction in depressive tendencies from BMAA practice was also independently mediated by both increased interoception of the Not-Worry dimension (the tendency not to raise emotional distress to discomfort) and decreased SoNA.

### 4.1 Discussion of the BMAA treatment effect

This study demonstrates that BMAA is an effective intervention for enhancing well-being among university students by decreasing their risk of depression. Furthermore, it enhances the entire spectrum of interoceptive sensibility, advancing from improved detection of internal signals to their effective use and the development of trusting beliefs in one’s body. Since interoception fundamentally supports emotional recognition and regulation, BMAA can be a powerful tool for cultivating these skills.

Notably, this is the first study to show that a short-term embodied intervention can improve SoA at a reflective level by reducing feelings of a lack of control over one’s actions or life. This finding has important clinical implications. First, it is especially relevant because 31.9% of the training group showed moderate to severe depression during the pre-test session. Since depression severity is inversely related to SoA as previously reviewed (Mehta et al., 2023; Vogel et al., 2024), a decrease in SoNA indicates that BMAA practice directly reduces the subjective experience of a temporary or lasting loss of agency—feeling unmoored or uncontrolled in one’s life—highlighting BMAA’s therapeutic potential.

Second, as previously noted, a low or negative sense of agency is common across various mental health disorders (e.g., Gallagher and Trigg, 2016; Tapal et al., 2017; Rabellino et al., 2018; Colle et al., 2020; Colle et al., 2023; Mehta et al., 2023; Vogel et al., 2024). This suggests that BMAA may offer broader therapeutic benefits beyond depression by targeting a core transdiagnostic construct, which warrants further exploration.

Contrary to the findings for SoNA, the BMAA practice did not enhance participants’ Sense of Positive Agency (SoPA). We suggest two plausible reasons for this discrepancy. First, the items of SoPA emphasize full, unrestricted control over what and when to act, which was severely restricted given that this study was conducted during Taiwan’s worst COVID-19 outbreak. The limited real-world opportunity for independent action likely constrained subjective measures of positive agency. Second, cultural factors may also play a role in the lack of SoPA enhancement. Several items on the SoPA scale emphasize an independent, context-free form of autonomy (e.g., “Things I do are subject only to my free will”), which may differ from the interdependent self-construal and the agency under situational constraints often emphasized in East Asian culture (Markus and Kitayama, 1991; see Chang et al., 2019, for a review). Consequently, the SoPA items may be less sensitive to our participants’ lived sense of agency. Other subjective measures of SoA that are more culture-fair need to be developed for examining this possibility.

### 4.2 The mechanism underlying BMAA’S effect on depression tendencies

Findings from the serial mediation analyses further reveal how different interoceptive sensibilities interact with SoNA and influence the BMAA’s effect on depression tendencies. First, Models 2, 4, and 8, which involve the Not-Distracting, Attention-Regulation, or Trusting dimensions, support our hypothesis and show that the embodied BMAA practice reduces depressive tendencies through a new, relatively bottom-up serial pathway. These findings align with the claim that SoA derived from interoception is crucial in clinical settings (van der Kolk, 2014) and contrast with previous findings that emphasize top-down cognitive strategies as the main mechanism linking interoception and emotion regulation in mindfulness-based interventions (Fissler et al., 2016; Garland et al., 2017).

Note that these three dimensions each reflect different levels of trust and connection to one’s body, regardless of comfort. They are not simply noticing bodily signals, nor are they explicitly using them to regulate emotions. Our results suggest that the influence of these interoceptive aspects on reducing depression operates through an increase in SoA. This may explain some paradoxical findings regarding the mediating role of these interoceptive aspects in distress alone. For example, as mentioned in the Introduction, evidence shows that participants who scored higher on the Not-Distracting dimension experienced more severe mental distress (Solano López and Moore, 2019). In contrast, our Model 2 indicates that the tendency not to ignore discomfort could be beneficial for depression when an individual’s ability to regulate bodily discomfort is also improved, as demonstrated by a decrease in a negative sense of agency.

Unlike the three interoception dimensions discussed earlier, each showing a sequential mediating pathway, Model 3 revealed a different pattern: only two simple indirect effects—one involving increased Not-Worry tendency toward bodily signals and the other involving decreased SoNA—independently predicted BMAA’s impact on depression. Compared to the Not-Distracting dimension, the Not-Worry dimension reflects not only a tendency not to avoid bodily signals but also greater confidence and acceptance in managing discomfort, leading to less worry about them. As the Not-Worry dimension and the non-judging facet of mindfulness have a moderate positive correlation (Mehling et al., 2012; Bornemann et al., 2015; Hanley et al., 2017), the simple mediating effect of Not-Worry suggests that BMAA could also reduce depressive tendencies by enhancing one’s confidence and acceptance of bodily discomfort, a more cognitive route similar to that of mindfulness interventions.

The independent, simple mediating pathway involving only SoNA found in Model 3 (also observed in Models 1, 5, 7, and 8) shows that SoNA’s predictive power does not necessarily stem from improvements in specific aspects of interoception. We suggest that this significant independent effect may be due to BMAA’s influence on reducing participants’ physical rigidity (as discussed in the Introduction), which could decrease the misalignment between intention and action—as indicated by lower SoNA—and result in less depression.

The other four dimensions of interoception do not show an indirect influence on the BMAA’s effect on depression tendencies. Despite showing the largest training effect among all facets and significant correlations with changes in both depression and SoNA, the Self-Regulation dimension of interoceptive sensibility—defined as the ability to down-regulate distress through attention to bodily sensations—did not emerge as a significant mediator. It neither explained the effect of the BMAA training on depression on its own nor as part of the sequential pathway through SoNA. The indirect effect via SoNA alone and the direct effect in Model 6 were also nonsignificant. One plausible explanation is that Self-Regulation and SoNA capture conceptually and empirically overlapping aspects of perceived control, which results in substantial shared variance between the two mediators. When multiple mediators are highly correlated, their regression coefficients become less precise, leading to inflated standard errors and reduced power for detecting unique indirect effects (MacKinnon et al., 2002; Hayes, 2022). Thus, the absence of significant individual paths should not be taken to mean that these variables play no role, but rather that their overlapping variance makes it difficult to statistically isolate their distinct contributions within a serial mediation framework.

Although significantly improved after the BMAA training, the remaining three models involving MAIA subscales—Noticing, Emotional Awareness, and Body Listening—show no serial or simple mediating effects on reducing depression. In each model, only the simple mediating effect through SoNA was significant. This indicates that simply being more aware of bodily sensations, whether passively or actively, and their connection to emotional states, is not sufficient to improve SoA or reduce depression severity. While these tendencies may serve as a foundational building block for other, more effective aspects of interoception, they do not, on their own, drive therapeutic outcomes.

### 4.3 Contributions and limitations

The contributions and implications of this study are summarized below. First, our findings imply that the 10-week BMAA curriculum benefits university students’ mental health by increasing their interoception and SoA in an educational context. This is particularly important given the concerning global rise in mental distress among young adults over the last decade.

Second, this is the first study to empirically show that individuals’ SoA can be improved through short-term embodied intervention. This has important clinical implications, as mentioned, because improving SoA is crucial for recovery in various mental illnesses. Future research should investigate this potential by recruiting a clinical sample.

Third, theoretically, we not only identify a distinct sequential mediating pathway involving specific aspects of interoception and SoNA, which differs from typical mechanisms seen in mindfulness interventions, but also, for the first time in an intervention setting, examine how different facets of interoceptive sensibility interact with SoNA to reduce depression. This evidence further clarifies the previously mixed findings about the relationship between interoception and mental distress.

Although conducting this study in an educational setting during the pandemic has important real-world implications, it nevertheless imposes limitations on the study design. First, to ensure an adequate sample size during the pandemic, our training cohort was split into two smaller groups across consecutive semesters. Nevertheless, this second cohort’s experimental context was quite similar to that of the first: they were students enrolled in the same course, taught by the same instructor, in the following semester under the same pandemic restrictions, and they also did not know the specific intervention content during course selection. Furthermore, our preliminary analyses indicated that both cohorts of the training group showed significant improvement in depressive tendencies, SoNA, and all dimensions of interoceptive sensibility compared to the control group, suggesting that the training effect of BMAA is quite robust.

Second, because this study was conducted within an educational setting, we did not use typical random assignment. Instead, we maintained blinding by keeping participants unaware of the specific intervention content assigned to each course time, which shared the same course name and instructor. For additional evidence of BMAA’s effectiveness, a study should be carried out outside an educational setting using a standard, fully randomized design.

## Data Availability

The raw data supporting the conclusions of this article will be made available by the authors, without undue reservation.
