# Supplementary material for: From the body to the mind: interoception and sense of agency as mechanisms of depression reduction in the Body–Mind Axial Awareness (BMAA)
Source: Front Psychol. 2026 Apr 7;17:1755698. doi: 10.3389/fpsyg.2026.1755698 (PMC13095796; doi:10.3389/fpsyg.2026.1755698)
Supplement: Supplementary file 1 [file Table_1.DOCX]

| **Supplementary Table 1** The 10-session BMAA program outline. | | | |
| --- | --- | --- | --- |
| **Session** | **Somatic Practice** | **Psychological Guidance** | **After-class Assignments** |
| **Week 1** | Foundational Awareness   - Cranial-Sensory Loosening: Practicing movements of the eyes, ears, nasal cavity, lips, and tongue to soften cranial tension. - Grounded Fulcrums: Ankle and metatarsal mobility practice to experience the ground as stable support during natural walking. - Sinking Breath: Lying-posture breathwork to facilitate experiencing weight offloading and "observing downward and inward." | Brief Course Introduction and The Barriers to Relaxation   - Explaining the core barriers: (1) lack of fulcrum, (2) volitional interference, (3) long-term bodily stagnation. - Understanding how BMAA principles address these somatic-psychological barriers. | - Journaling: Documenting somatic feelings during practice and one’s reflections afterward in a personal online journal. - Daily Routine Application: To feel one's own and observe others’ habitual tension in the face and shoulders during daily computer use. |
| **Weeks 2–9** | Deconstructed Exploration and Axial Integration (under the same principle)   - Practicing diverse subtle mobilization with different positions as fulcrums to loosen and explore facial and other body parts, including the limbs (and joints), shoulders, scapula, chest, spine, and pelvic cavity. - Reinforcing the central axis through diverse postures and breath regulation. - Integrating practices to facilitate the sense of the inner pathway within a movement, from grounded fulcrums through the central axis to the terminal extremities across various postures. | - Viewing each movement as a process of self-exploration rather than a performance to be evaluated. - Practicing with "least intention" rather than forcing the body to meet a predetermined standard. - Perceiving discomfort as feedback on stagnation and observing its changes over time, rather than intentionally escaping or diminishing it. - Explaining how bodily fluidity facilitates emotional stability and fosters a sense of self-mastery. | - Journaling: Documenting somatic feelings during each movement and one’s reflections afterward, as practiced in Week 1. - Daily Routine Application:   - Finding opportunities to maintain the body-mind axial state in silence during activities such as walking, eating, commuting, or visiting nature and art exhibitions.   - Observing and adjusting one's workstation setup (desk, chair) to better sustain the body-mind axial state based on internal somatic feedback. |
| **Week 10** | Final Integrated Review   - Review and combine practices engaging different body parts and postures to learn the smooth transition and integration as a whole - To maintain a body-mind axial state while moving. | Introducing the traditional perspective of the ideal body-mind state：   - Tranquility Within, Reverence Outward - The approach to the ideal state is based on an unobstructed and flexible body. | No assignment. |

Note: Each weekly session lasts 180 minutes. In addition to the Somatic Practice (120 min) and Psychological Guidance (30 min) detailed above, each session concludes with a Group Discussion (30 min). While the BMAA program follows a structured progression from peripheral mobilization to central axis integration, the curriculum remains adaptive; the instructor may adjust the sequence or focus of specific exercises based on participants' real-time somatic feedback and individual progress.
